# Oral Manifestations in Melanoma Patients Treated with Target or Immunomodulatory Therapies

**DOI:** 10.3390/jcm10061283

**Published:** 2021-03-19

**Authors:** Emi Dika, Martina Lambertini, Bruna Gouveia, Martina Mussi, Emanuela Marcelli, Elena Campione, Carlotta Gurioli, Barbara Melotti, Aurora Alessandrini, Simone Ribero

**Affiliations:** 1 Division of Dermatology, IRCCS—Policlinico Sant’Orsola, via Massarenti 9, 40138 Bologna, Italy; carlotta.gurioli@gmail.com (C.G.); barbara.melotti@aosp.bo.it (B.M.); aurora.alessandrini3@unibo.it (A.A.); 2 Division of Dermatology, Department of Experimental, Diagnostic and Specialty Medicine (DIMES), University of Bologna, 40138 Bologna, Italy; mlambertini@hotmail.it (M.L.); mussi.martina1809@gmail.com (M.M.); 3 The Poche Centre, Melanoma Institute of Australia, 40 Rocklands Rd, Wollstonecraft, NSW 2065, Australia; bruna.gouveia@melanoma.org.au; 4 Laboratory of Bioengineering, Department of Experimental, Diagnostic and Specialty Medicine (DIMES), University of Bologna, 40138 Bologna, Italy; emanuela.marcelli@unibo.it; 5 Dermatology Unit, University of Rome Tor Vergata, 00133 Roma, Italy; campioneelena@hotmail.com; 6 Department of Medical Sciences, Dermatologic Clinic, University of Turin, 10126 Turin, Italy; simone.ribero@unito.it

**Keywords:** melanoma, oral, adverse event, target, immunotherapy

## Abstract

Background: BRAF (v-raf murine sarcoma viral oncogene homolog B1) and MEK (mitogen activated protein kinase) inhibitors, as well as immunotherapy against cytotoxic T-lymphocyte-associated antigen 4 (CTLA-4) and the programmed cell death 1 (PD-1) receptor and its ligand (PD-L1), have shown good results in improving the disease-free survival of patients with metastatic melanoma (MM). The aim of this review is to summarize the main oral adverse events (oAEs) occurring in patients undergoing target or immunotherapy. We proposed two separate sections: oAEs during the treatment with (1) target therapies with BRAF and MEK inhibitors and tyrosine kinase inhibitors (gingival hyperplasia, pigmentation disorders, squamo-proliferative lesions) and (2) immunotherapies with CTLA-4 or PD1 inhibitors (lichenoid reactions, immuno-bullous reactions, xerostomia and other reactions). Adverse events frequently include oAEs, although these are often misdiagnosed and under-reported. Indeed, the oral cavity is not routinely evaluated during clinical practice. The symptomatology related to oAEs is significant since it may represent the first manifestation of a severe systemic reaction, possibly leading to difficulties in nutrition with a consequent impact on patients’ quality of life. A careful examination of the oral cavity is recommended during the evaluation of oncologic patients in order to promptly detect the onset of new manifestations.

## 1. Introduction

**Figure 1:**
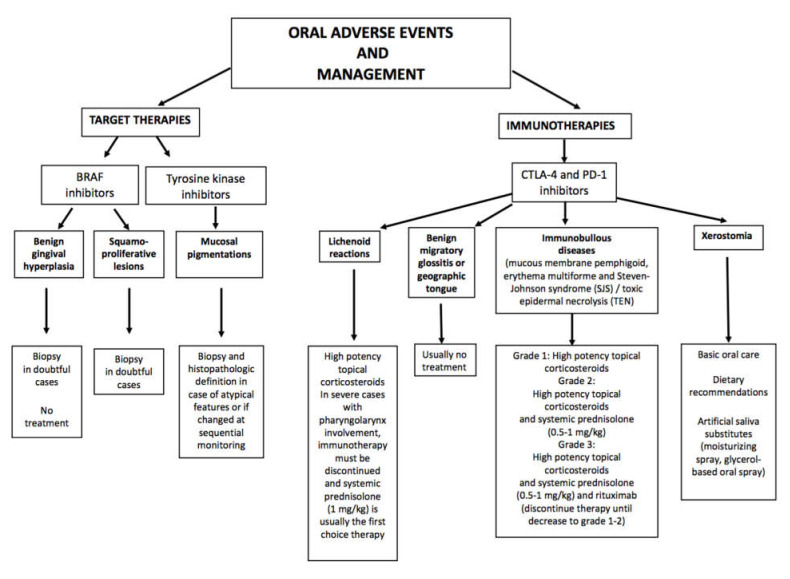
Oral adverse events and their management occurring during the treatment with target therapies (BRAF inhibitors and tyrosine kinase inhibitors) and immunotherapies (CTLA-4 and PD-1 inhibitors). BRAF (v-raf murine sarcoma viral oncogene homolog B1); CTLA-4 (checkpoints T-lymphocyte-associated protein 4); PD-1 (programmed cell death protein 1).

Target and immunomodulatory drugs have proved to be very promising in patients with metastatic melanoma (MM) [1]. BRAF (v-raf murine sarcoma viral oncogene homolog B1) and MEK (mitogen activated protein kinase) inhibitors, as well as immunotherapy targeting cytotoxic T-lymphocyte-associated antigen 4 (CTLA-4) and the programmed cell death 1 (PD-1) receptor and its ligand (PD-L1), have shown good results in improving the disease-free survival of MM patients [1]. However, the development of cutaneous toxicity has been frequently reported in the course of both target and immunotherapy. Approximately 30–40% of treated patients with target and immunotherapy report cutaneous adverse events (cAEs), respectively [2]. Adverse events frequently include oral manifestations (oAEs), although these are often misdiagnosed and under-reported (Figure 1) [2]. Indeed, the oral cavity is not routinely evaluated during clinical practice. However, the symptomatology related to oAEs may be significant, determining difficulties in nutrition and a heavy burden for the patient. OAEs may even represent the first manifestation of a severe systemic reaction.

The aim of this paper is to summarize the main oAEs occurring in patients undergoing target or immunotherapy. The recognition and definition by the clinician of oAEs is crucial in optimizing patient care.

## 2. Materials and Methods

The present critical review of the literature evaluated the oAEs associated with the use of immunotherapies or targeted therapies for the treatment of MM.

The review process was conducted and reported from the following databases:

- PUBMED
- Ovid MEDLINE

The literature review was conducted from 2011 (the year of approval by the Food and Drug Administration of the BRAF-inhibitor Vemurafenib) to June 2020.

The keywords and/or MESH terms used were: “melanoma”, “target therapy”, “immunotherapy”, ”oral pigmentation”, “oral adverse events”, “oral reactions”, “bullous manifestations”, “cutaneous adverse events”. Additional studies were found through cross-referencing of reference lists from the retrieved articles and previous reviews on the topic. 

The analyzed papers included original articles, reviews, and case reports (due to the frequently limited number of patients).

Data were extracted from each paper on (a) characteristics of the oral event, (b) final diagnosis, (c) pathogenetic mechanism, (d) proposed treatment, (e) outcome.

We proposed two separate sections: oAEs during the treatment with (1) target therapies with BRAF and MEK inhibitors and tyrosine kinase inhibitors and (2) immunotherapies with CTLA-4 or PD1 inhibitors.

Studies reporting oral adverse events in patients with other types of cancer were excluded.

## 3. Target Therapies with BRAF and MEK Inhibitors and Tyrosine Kinase Inhibitors

### 3.1. Gingival Hyperplasia

**Figure 2:**
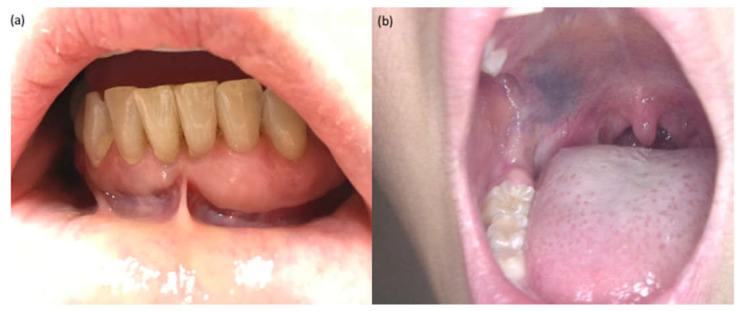
Clinical presentation of (**a**) benign gingival hyperplasia in a patient in treatment with a BRAF inhibitor (vemurafenib); (**b**) a pigmentation of the hard palate induced by imatinib.

In the oral cavity, multifocal hyperkeratotic mucosal lesions, mainly arising on the free edge of the gingiva, the palate, linea alba, the lateral sides of the tongue, or the lining mucosa of the lips, have been reported in relation to BRAF inhibitor therapy [1]. A verrucous or papillomatous surface is usually observed and the disorder is defined as “benign gingival hyperplasia” (Figure 2a) [2,3]. The hypothesis of a possible side effect of RAS/mitogen-activated protein kinase activation has been advanced. Similarly, patients affected by rasopathic syndromes such as Noonan syndrome-4, and hereditary gingival fibromatosis-1 may present congenital anomalies and ectodermal changes that encompass gingival hyperplasia and gingival fibromatosis [4,5,6]. Dental abnormalities have not been reported during the treatment with targeted therapies.

A careful oral evaluation is not routinely performed in treated patients and consequently the real incidence of oral BRAF induced mucosal hyperkeratosis remains unknown. Oral squamous cell carcinomas (SCCs) are uncommon during targeted therapies, but a biopsy should be recommended in doubtful cases. Nowadays BRAF inhibitors are usually prescribed in association with MEK inhibitors (vemurafenib+cobimetinib, dabrafenib+trametinib and encorafenib+binimetinib) in order to block the mitogen-activated protein kinase (MAPK) pathway and reduce the occurrence of hyperkeratotic lesions [7,8,9,10].

### 3.2. Pigmentation Disorders

The most remarkable cutaneous adverse events related to KIT inhibitors, especially imatinib, are alterations of skin, hair and mucosal pigmentation. Blue-brownish-black hyper-pigmentations of the palate have been described during the treatment with imatinib (Figure 2b) [11,12,13]. KIT inhibitors are small-molecule tyrosine kinase inhibitors. For imatinib, cutaneous, mucosal and hair pigmentation alterations are thought to be related to a paradoxical gain-of-function of the c-Kit pathway, inducing melanocytes proliferation and consequent melanocytic hyperplasia. A positive correlation was shown between the onset of the pigmentations and the duration of imatinib treatment [12,14]. Biopsy and histopathologic definition should be limited to focal hyperpigmented lesions with atypical features or those evolving at sequential monitoring. Clinical and dermoscopic follow-up is recommended [15,16].

### 3.3. Squamoproliferative Lesions

Vemurafenib, the first introduced BRAF inhibitor drug, was associated with a multitude of cAEs, including rash, increased sensitivity to ultraviolet rays, palmar-plantar erythro-dysesthesia (“hand-foot syndrome”), alopecia, itching, keratosis pilaris-like manifestations and hyperkeratosis [17,18,19,20,21,22,23,24]. Most cAEs were generally considered grade 1–2, mild to moderate and well tolerated [25]. The most worrisome features related to cAEs included the occurrence of acanthopapillomas (AP), actinic keratosis (AK), keratoacanthomas (KA), and SCC [26,27]. BRAF inhibitors can reversibly bind to the mutant *BRAF*V600E to block the downstream extracellular signal-regulated kinase (ERK) pathway, though contrasting effects are detected in wild-type cells or in cells with UV-induced mutant *RAS*, secondary to the heterodimerization of BRAF kinases. RAS pathway paradoxical activation, detected in up to 1/5 of subjects treated with BRAF inhibitor monotherapy, determines cutaneous hyperkeratotic lesions, including SCC and KA [26,28,29]. Most squamo-proliferative lesions (SCC, KA, AP) develop in an eruptive way, as raised and hypertrophic keratotic papules [26,28,30]. A particular observation on histopathology was the similarity of AP features to those of verrucae vulgares, showing conspicuous hyperkeratosis with parakeratosis in the absence of koilocytes. Studies assessing the presence of HPV genotypes in AP and SCC biopsies resulted negative [28]. Mutational analyses of the RAS family genes (*HRAS*, *NRAS*, *KRAS*) from BRAF inhibitor-induced lesions with a histopathologic diagnosis of KA or SCC were implemented in various studies, showing a benign course for most lesions, except for rare cases with *RAS* mutations, mostly *HRAS*, associated with an aggressive behavior [26,28]. Distant recurrences and metastatic disease related to BRAF inhibitor-related SCC have not been reported so far. The occurrence of mucosal AP or SCC is rare. A single case of hyperkeratotic neoplasms of the labial mucosa was reported by Vigarios et al. in a patient treated with BRAF-inhibitor [31].

## 4. Immunotherapies with CTLA-4 or PD1 Inhibitors

Use of CTLA-4 and PD1 or PDL-1 inhibitors, in monotherapy or in association with other agents, determines a specific range of AEs, mainly due to the altered immune response, with cAEs being the most common. Therapy with agents blocking PD-1 activity (nivolumab, pembrolizumab) is related to stomatitis or oral inflammation in a few mild to moderate cases (grade < 3). Specific mucosal alterations have been reported during the treatment with PD-1 inhibitors, including lichenoid and immuno-bullous, plus some unclassifiable reactions. Moreover, a very common disease impacting on the patients but without a specific dermatologic feature is xerostomia.

### 4.1. Lichenoid Reactions

Oral lichenoid reactions (OLR) can occur either due to intake of drugs or due to contact with specific molecules such as those used for dental treatments [32]. In 2016, Schaberg et al. firstly reported the clinicopathological description of lichenoid reactions related to anti-PD-1 and anti-PD-L1 therapies [33]. In our practice, this subtype of lesions is relatively common but is likely to be underestimated due to the usual lack of symptoms. The diagnosis is often fortuitous. In the literature, it has been reported that 3.8% of patients on anti-PD1 therapy present cutaneous and OLRs [34]. Women experience a two to three times higher rate of OLRs compared to men, with a peak in the fifth decade of life [32,35]. It is important to exclude other causes before associating the lichenoid reaction to the immunotherapy. The possible risk of neoplastic degeneration of OLR is estimated to range between 0.4% and 6.5%, mostly <1%, so a periodical follow-up is suggested [3,36]. “Atypical” clinical presentations, including atrophic and erosive-ulcerative subtypes, are likely to be related to a higher chance [37].

**Figure 3:**
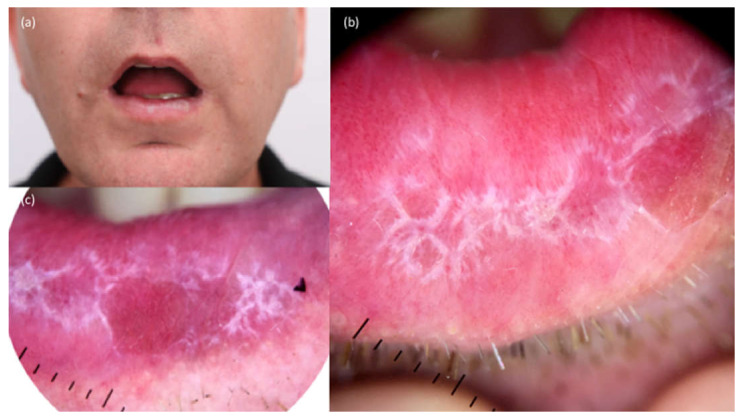
Clinical presentation (**a**) of an oral lichenoid reaction showing reticular features and Wickham’s striae (**b,c**) in a patient treated with Anti-PD1 (nivolumab).

Unilaterality is a common feature of ORLs, whereas oral lichen planus usually progresses to a bilateral and symmetrical distribution [32,35]. On physical examination, it is possible to identify whitish and reticulated plaques (Wickham’s striae) mainly located on the buccal mucosa, palate and inner portion of the lips (Figure 3). Polymorphic features are common, showing an association of reticular/striated lesions with symptomatic erosive or atrophic elements. Oral lesions can be a single finding or associated with nail plate or cutaneous abnormalities [3]. The onset has been reported even several months after the first immunotherapy dose [3]. However, one case of OLR was reported to occur after the second dose of nivolumab [34]. In our practice, patients undergoing nivolumab have developed oral and cutaneous lichenoid reaction in the third cycle [34].

Anti-PD1-associated symptoms mainly arise on the buccal or lingual mucosa, with pain being the most commonly reported [36]. Taste disorders or xerostomia are other possible sensations experienced by the patients [32]. Enomoto et al. [36] reported a grade 3 OLR associated with pharyngo-laryngitis in a subject in treatment with nivolumab. In cases with pharyngo-larynx involvement, sometimes arising before mucosal alterations, there is a risk of respiratory stenosis and early detection and management are mandatory. Histology shows interface dermatitis with a lymphocytic infiltrate in the dermo-epidermal junction together with basal and supra-basal apoptotic keratinocytes [3]. A more prominent spongiosis is usually detected in drug-induced OLRs compared with bioptic samples of other subtypes of lichenoid reactions [33]. It is noteworthy to point out that subjects on treatment with anti-PD1 may result positive for anti-BP180 autoantibody with no immuno-bullous reaction. A potential explanation for this positivity is related to BP180 expression not only on the basement membrane zone but even on the surface of melanoma and nonsmall-cell lung carcinoma cells. The blockage of the PD-1/PD-L1 signaling pathway may enhance autoantibody development targeting the BP180 with the mediation of both T- and B-cells [36]. The possible evolution of OLRs to a bullous form is unpredictable. Direct immunofluorescence can be performed to rule out bullous pemphigoid [36].

High potency topical corticosteroids are usually the first line treatment option and immunotherapy are not discontinued in the majority of patients. If lesions are multiple or in case of a severe involvement, oral steroid may be recommended. The prompt identification of the disease and the correct treatment may prevent immunotherapy interruption and improve patients’ quality of life. For severe cases with pharyngo-larynx involvement, immunotherapy must be discontinued and systemic prednisolone (1 mg/kg) is usually the first choice therapy. Oral symptoms usually heal within the weeks after the beginning of the therapy [36].

### 4.2. Immunobullous Reactions

**Figure 4:**
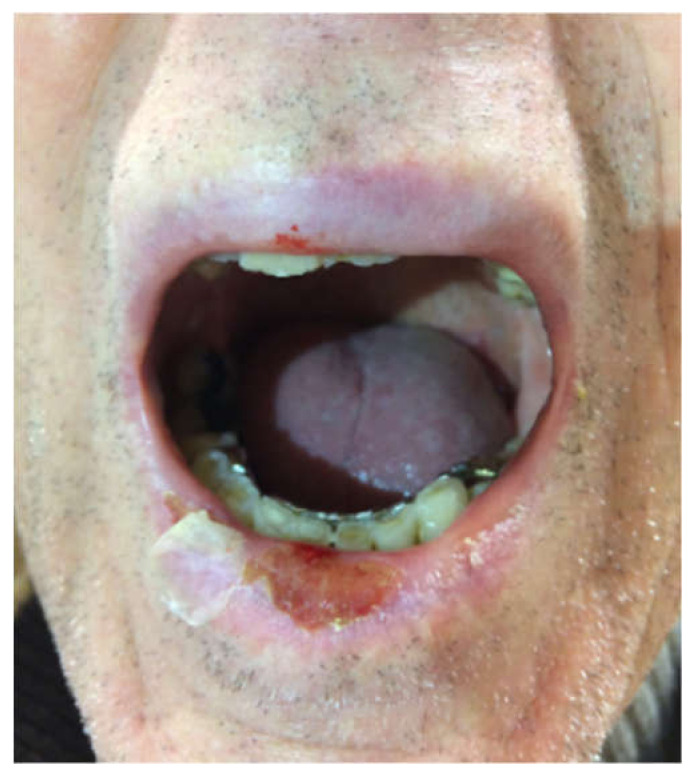
Oral involvement of a bullous pemphigoid in a patient in immunotherapy with Anti-PD1 (nivolumab).

Immuno-bullous diseases have been reported in pembrolizumab and nivolumab patients, as well as in those under anti-CTL-4 agents [38]. A few cases of mucous membrane pemphigoid during anti-PD1 treatment have been reported. It has been hypothesized that anti-PD1 antibody enables preexisting or new autoimmune T cell clones to evade the immune system under the control of regulatory T cells. Alternatively, it has been described that the PD1-block on B cells can increase antigen-specific antibody responses [39,40]. Vesiculobullous rashes are possible manifestations related to anti-PD-1 treatment, with the risk of rapid evolution and relevant morbidity. No cases of exclusively mucosal bullous pemphigoid have been reported, but the oral mucosa can be involved in 10–30% of cases with cutaneous involvement (Figure 4) [41]. Hemi-desmosomal structural proteins of the dermo-epidermal junction, BP180 (collagen XVII), and BP230, are targeted in BP. The detection by ELISA of circulating autoantibodies against BP180 and BP230 is a diagnostic test to confirm the diagnosis, to assess disease severity, or evaluate the therapeutic effect. BP with anti-PD-1/PD-L1 mAbs may develop even many months after the beginning of the therapy, in association or preceded by an itching sensation.

There are no peculiar elements to distinguish classic BP from drug-induced BP. Drug-induced BP generally heals after the discontinuation of the causative agent, but a chronic disease similar to classic BP may be observed [42,43].

**Figure 5:**
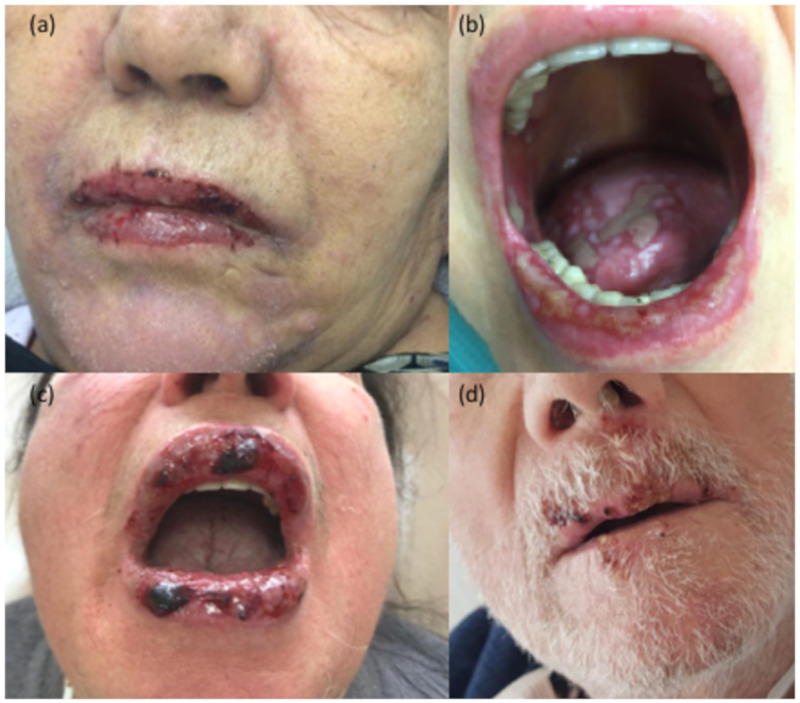
Steven-Johnson syndrome in patients in treatment with immunotherapy with Anti-PD1 (nivolumab and pembrolizumab) affecting the lips (**a,b,c,d**) or the tongue (**b**).

The involvement of oral mucosa can be present in erythema multiforme and Steven-Johnson syndrome (SJS)/Toxic epidermal necrolysis (TEN) as a further manifestation of the syndrome (in 80% of cases) [41], especially when two immunotherapies are combined [44] (Figure 5). In half of the cases a prodromal rash is reported before the onset of oral/conjunctival/genital abnormalities and cutaneous blistering. Histopathology of TEN/SJS reveals full-thickness necrosis of keratinocytes. PDL1 immunohistochemistry was performed in the inflammatory infiltrate of one TEN case and one SJS case, and both resulted as highly positive [45].

### 4.3. Xerostomia

Xerostomia has been described in up to 6% and 7.2% of MM subjects treated with nivolumab and pembrolizumab, respectively. It is the second most common oAE and it is sometimes accompanied by dysgeusia [3]. It is usually graded as mild (grade 1–2), but it can be extremely severe. Clinical elements of Gougerot-Sjögren-like syndrome, with cytotoxic T-lymphocyte infiltration of accessory salivary glands, are found, though anti SSA and anti SSB antibodies are not detected. Indeed, the term “sicca syndrome associated with immune checkpoint inhibitor therapy” has been proposed [46]. The onset is quite sudden and patients progressively need to drink a greater amount of water in order to chew and swallow dry foods. The symptoms tend to worsen with physical activity or at night-time. Other diagnostic elements are thick or sticky saliva, throat dryness with hoarseness, dysgeusia, and reduced tolerance to spices or acid foods. Clinical alterations due to salivary gland hypofunction are found in most of the subjects, including chronic erythematous candidiasis as well as ulcerative lesions and/or burning sensation. Accurate oral hygiene, dietary advices and the use of over-the-counter saliva substitutes (moisturizing spray, glycerol-based oral spray) should be prescribed to reduce the burden daily life.

### 4.4. Other Reactions

**Figure 6:**
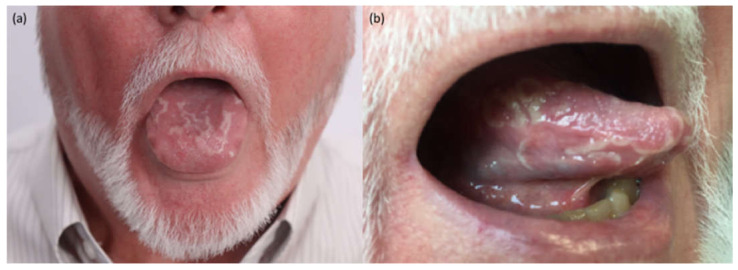
Clinical presentation of a benign migratory glossitis or geographic tongue after the first cycle of nivolumab affecting the dorsal (**a**) and the lateral (**b**) surfaces of the tongue.

Benign migratory glossitis, also known as geographic tongue, is an oAE primarily observed during the treatment with antiangiogenic targeted therapies, bevacizumab [47], sorafenib and sunitinib [48,49,50]. It is also observed with pazopanib and axitinib. In our experience, geographic tongue may even occur with anti-PD1 drugs, but the real impact is still unknown [49]. Figure 6 shows a patient who developed this symptom after the first cycle of nivolumab with an intermittent course. A moderate pain can be experienced by the patient, but it usually does not determine any therapeutic modification. The mucosal abnormalities usually disappear after treatment interruption.

## 5. Study Limitations

Our study has some limitations. Firstly, we did not perform a systematic review or a meta-analysis due to the difficulties in obtaining raw data from previous studies and the frequent limited number of patients, moreover due to the multi-disciplinary approach in the management of oral diseases, including the collection of clinical images and patients’ data. Finally, this critical review included a specific focus on oral adverse events occurring during the treatment with target therapies or immunotherapies for metastatic melanoma, rather than other types of cancer.

## 6. Conclusions

In conclusion, oAEs are often misdiagnosed and under-reported but they may occur during target or immunomodulatory therapies for MM, or in the setting of an adjuvant therapy. A careful examination of the oral cavity is recommended during the evaluation of oncologic patients in order to promptly detect the onset of new manifestations. Indeed, oAEs may be the first manifestation of a severe systemic reaction, possibly leading to difficulties in nutrition with a consequent impact on patients’ quality of life

## Data Availability

Data sharing not applicable.
